# Assessing the Small Nuclear Ribosomal Subunit (18S rRNA) as a Potential Barcoding Gene for Amphipoda (Malacostraca: Peracarida) Along with the Design of a Unique Primer Set to Target the Amphipod 18S rRNA Gene

**DOI:** 10.3390/biology15191752

**Published:** 2026-10-02

**Authors:** Thomas R. Sawicki, Andrew G. Cannizzaro, Joshua M. Sisco

**Affiliations:** 1 Department of Biological Sciences, Florida A&M University, Tallahassee, FL 32307, USA; joshua1.sisco@famu.edu; 2 Department of Biology, Miami University, Oxford, OH 45056, USA; cannizag@miamioh.edu

**Keywords:** cryptic species, phylogenetics, Crustacea, COI

## Abstract

The mitochondrial cytochrome oxidase I (COI) gene is frequently used as a barcoding gene for the identification of numerous taxa, including crustaceans such as amphipods. The gene’s variability, which makes it useful for barcoding, can also create primer annealing issues that can inhibit amplification and sequencing, and nuclear copies of mitochondrial DNA (NUMTs) can sometimes preferentially amplify over the mitochondrial COI target locus. The small nuclear ribosomal gene (18S rRNA) can overcome issues of amplification and sequencing as primers can be designed to target highly conserved regions, making it useful across a wide variety of taxa. Due to its conserved nature, the 18S rRNA gene has not been considered a strong candidate for barcoding. Peracarid crustaceans have an insertion within the V4 hypervariable region, and amphipods are unique amongst the peracarids with an additional insertion in the V5 hypervariable region, which could make this part of the gene large and variable enough to target for barcoding. Herein, we present three primer sets designed for Amphipoda that amplify the majority of the 18S rRNA gene, including one pair (18S450F-18S1500R) that captures the V4/V5 hypervariable regions. Analyses utilizing tree-building algorithms and two species delimitation models demonstrate the potential utility of the gene region amplified by the 18S450F-18S1500R primers for barcoding. The utility of this gene for barcoding will depend on researchers incorporating this locus into future species descriptions and phylogenetic studies.

## Simple Summary

Traditionally, determining the taxonomic identity of an organism has required detailed morphological examination by highly skilled taxonomists. However, genetic data have revealed substantial hidden diversity, including cryptic species that are genetically distinct despite being morphologically very similar. As genetic databases have grown, scientists have increasingly used DNA sequences to identify species through a process known as DNA barcoding. The mitochondrial cytochrome oxidase I (COI) gene is commonly used for this purpose, but its high variability can complicate amplification and sequencing, and nuclear copies of mitochondrial DNA can sometimes produce misleading sequences. The nuclear 18S ribosomal RNA gene is easier to amplify across diverse organisms but is generally considered too conserved for species-level identification. Amphipod crustaceans, however, possess unusually large variable regions within the 18S gene. Here, we identify an approximately 500–600 base pair amphipod region of hypervariability and develop new primers to amplify the 18S gene, including this region. Our analyses demonstrate that this hypervariable region can differentiate closely related amphipod species, supporting its use as a potential complement to COI for amphipod species identification and future taxonomic studies.

## 1. Introduction

Amphipoda is one of the most ecologically diverse and speciose orders of malacostracan crustaceans, composed of over 10,000 species divided into six suborders [1,2,3]. In marine environments, amphipods occur from polar to tropical regions, and in extraordinarily diverse habitats, ranging from littoral to hadal zones, with taxa occurring as deep as ‘Challenger Deep’ within the Mariana trench [4,5,6]. Amphipods display similar diversity in freshwater systems, with taxa occurring in nearly all forms of freshwater environments, including lakes, rivers, streams, marshes, springs, seeps, and caves; in hypogean systems like caves, amphipods are often some of the most abundant taxa recorded [7,8,9,10].

As members of the superorder Peracarida, amphipods brood their young and lack a larval stage capable of long-range dispersal [8,10]. Due to this fact, along with their generally small size (usually between 2 and 20 mm), amphipods possess intrinsic barriers to dispersal not found in other taxa; these can result in considerable restrictions to gene flow across broadly distributed metapopulations [11,12]. Significant work has been conducted in recent years demonstrating high levels of cryptic diversity within amphipod species in a variety of environments, both freshwater and marine, as well as epigean and hypogean [11,12,13,14,15,16,17]. Despite representing distinct molecular lineages, cryptic species often display little to no morphological differentiation, which can result in even taxonomic experts incorrectly identifying a species where identity is based on morphology alone [18].

Aside from morphological examination, a common way of identifying species is via ‘molecular barcoding’, where individuals are sequenced at a specific locus and then compared with a reference database, such as GenBank. One of the most common loci used for barcoding across most metazoan taxa is the mitochondrial cytochrome oxidase subunit I (COI [12,19,20,21]). Despite its ubiquity in both barcoding and in phylogenetic studies, use of the COI locus can present some issues, including the following: (I) due to their hypervariable nature, primers occasionally fail amongst closely related taxa or even across individuals of the same species collected from the same location requiring the production of custom internal primers [17,22,23,24]; (II) the presence of nuclear copies of mitochondrial DNA (NUMTS [25]), which can either prevent sequencing due to the amplification of both target and NUMT sequences or produce pseudogene sequences [26,27,28]; (III) amplification using ‘universal’ primers, such as the commonly used LCO1490/HCO2198 primers [29], will sometimes amplify non-target DNA, leading to the sequencing of ‘contaminant’ taxa such as prokaryotes or fungi instead of the intended organism [30,31].

Due to the presence of hypervariable regions (particularly the V4 and V7 in which large insertions often occur), the nuclear small-subunit ribosomal RNA gene (18S rRNA) varies significantly in length across metazoan taxa [32] with range lengths varying among peracarids from approximately 1800–2900 base pairs [33,34]. The length of the gene along with the high degree of sequence conservation among the hypervariable regions has resulted in this locus being commonly used for phylogenetic analyses, particularly to assess relationships among deeply divergent taxa [35,36,37]. Due to its highly conserved nature, the gene is often considered incompatible, or at best weakly compatible, for species-level identification [38,39,40]. Despite this, it has been documented to be effective at species delimitation across a variety of amphipod families including leucothoids [41,42], parabogidiellids and crangonyctids [43,44] and gammarids [12,16]. This utility is, in large part, due to the presence of an insertion within the V4 hypervariable region unique to peracarids (excluding Mysida, Lophogastrida and Mictacea), with amphipods unique amongst peracarids in possessing an additional insertion in the V5 hypervariable region [33]. Among Amphipoda, the V4/V5 hypervariable regions occur in proximity within a region of approximately 700–1200/1300 base pairs along the transcript. This approximately 500–600 bp region, which from hereon we call the AROH (amphipod region of hypervariability), has been demonstrated to be able to delimit even closely related amphipod species [18].

Unlike the common ‘universal’ primer sets utilized for COI, several 18S rRNA primer sets have been designed for various metazoans [45,46,47]. For peracarids, a primer set designed by Spears et al. [33] is available; however phylogenetic studies for Amphipoda commonly use primers first published by English & Koenemann [48] (see [15,44,49]). The Englisch & Koenemann [48] primers were not designed specifically for amphipods (Englisch, personal communication) and periodically fail to produce amplicons and/or quality sequences, particularly when attempting to capture the AROH, due to the increased variability in this region. To remedy this, we present three novel primer sets designed to sequence ~2100 base pairs of the 18S rRNA for nearly all amphipod crustaceans, with one of the three sets specifically targeting the AROH (and additionally encompassing the much less variable V3 and V6 regions). This primer set produces a sequence of approximately 900 base pairs that may be useful as a barcoding gene for species within the order. Herein, we test the potential use of this locus for barcoding.

## 2. Materials and Methods

### 2.1. Sampling and Morphological Examination

Individuals collected as a part of this project were either obtained using dip/hand nets or with a ‘Sket bottle’ [50] while SCUBA diving (as was the case for the population of *Gammarus minus*, Say, 1818, from Scherr Spring). All specimens were preserved in 100% molecular-grade ethanol and stored at −30 to −80 °C (Table 1). Initial morphological identification was conducted by comparing diagnostic characteristics and/or utilizing dichotomous keys in Bousfield [4,51] and Lecroy [5,52,53].

### 2.2. Developing a Reference Database and Primer Design

**Figure 1:**
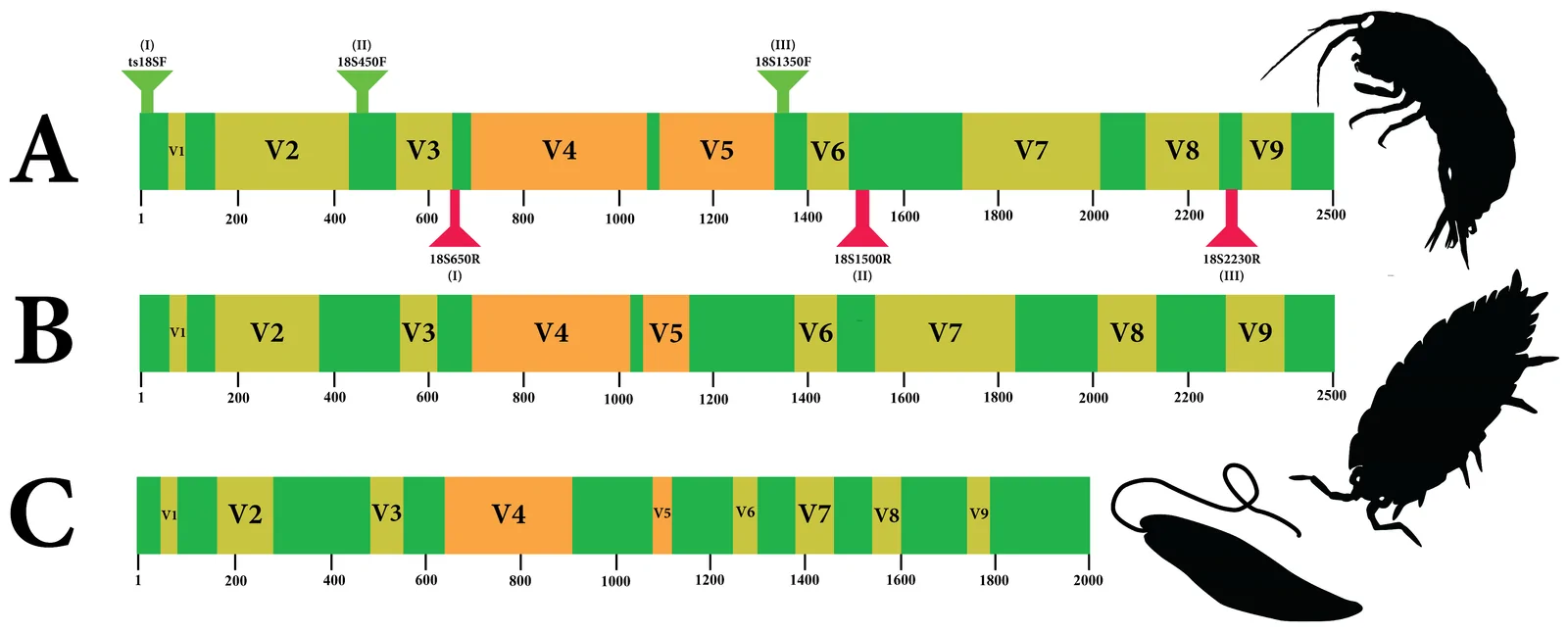
Relative positions of hypervariable regions in the 18S rDNA among selected taxa placed on linear maps: (**A**) Amphipoda: positions of newly designed primers are also indicated above and below the map; (**B**) non-amphipod peracarids; (**C**) non-peracarid eukaryotes, modified from [45]. For all taxa, the V4 and V5 hypervariable regions are indicated in a different color.

A local database of 18S rRNA sequences was developed by querying GenBank for members of Amphipoda (Table 2). Of the sequences present within the GenBank database, only sequences with a minimum length of 2300 base pairs were retained and used for primer development. In addition to amphipod sequences, *Ingolfiella tabularis* Stock, 1977, (Order: Ingolfiellida) was also included due to the high levels of similarity among these orders. Sequences were aligned using MAFFT (v.7.388 [54,55]) in GENEIOUS PRIME (v.10.2.3; www.geneious.com [56]) and the ‘Geneious Alignment’ tool in GENEIOUS PRIME. Primers were designed using the ‘Design New Primers’ tool (Primer 3 [57]) available in GENEIOUS PRIME and further modified by eye. To capture most of the gene, three primer pairs were designed based on the following criteria (see Figure 1 for primer annealing sites):

(1) Primer size ranges between 18 and 25 base pairs, with the optimal set at 20;
(2) Tm ranges between 57 °C and 64 °C, with the optimal set at 60 °C; %GC range between 20 and 80, with the optimal set at 50;
(3) Primers are 100% conserved across most taxa used for the design—maximum mismatches of four base pairs across the entire primer, with 0 mismatches within the last six base pairs;
(4) GC clamp (or sequence ending with a G or C) on the 5′ and 3′ ends of each primer;
(5) Annealing temperatures within 5 °C for each pair of primers;
(6) For the optimal downstream sequencing, the products of each primer pair of approximately 1000 base pairs or less;
(7) Primer pair products with sufficient overlap so that alignments of each section produce contiguous sequences.

The three resultant primer pairs (ts18SF-18S650R; 18S450F-18S1500R; and 18S1350F-18S2230R) (see Table 3) produced amplicons ranging in length from approximately 650 to 900–1000 base pairs in length.

### 2.3. In Silico Testing

The three generated primer pairs (Table 3) were analyzed for secondary structures (hairpins and primer-dimers) and for self-complementarity using the online Oligonucleotide Properties Calculator v. 3.27 [58]. Each primer set was tested against the SILVA SSU r138.2 Database [59], with no mismatches allowed, to determine its ability to anneal with amphipod sequences. Primer sequences demonstrated a high degree of conformity across all species used to generate the primers, with the most mismatches exhibited among hyperiid species (4 across the 22 base pairs) in the ts18SF primer. All other species exhibited only a single mismatching base pair across all designed primers, and the 18S1350F primer was 100% conserved across all taxa analyzed (Table 4).

### 2.4. Empirical Testing—PCR Preparation, Thermal Cycler Protocols, and Sequencing

Primers were tested on species from a variety of amphipod families to ensure efficacy of both the amplification and Sanger sequencing (Table 1). Genomic DNA extractions were performed using the Quick-DNA™ Miniprep Plus Kit (Zymo Research, Irvine, CA, USA) following the modification of the manufacturer’s protocol. Whole-body specimens were used for DNA extraction. In each extraction, 85 µL of molecular-grade water, instead of the recommended 95 µL, 95 µL Solid Tissue Buffer, and 20 µL Proteinase K, instead of the recommended 10 µL, were used. In cases where the tissue sample was not completely submerged in the prepared lysis solution, the volumes of all lysis solution components were doubled. Samples were incubated at 55 °C for approximately 4 h until the internal tissues were largely dissolved, instead of the 1–3 h incubation period specified in the manufacturer’s protocol (the exoskeleton remained intact after digestion). Following incubation, lysates were transferred to clean microcentrifuge tubes and the purification steps were carried out according to the manufacturer’s protocol. Eluted DNA was stored at −20 °C.

PCR reactions consisted of 12.5 μL APEX Red 2X Master Mix DNA Polymerase (Genesee Scientific, El Cajon, CA, USA), 1 μL of each primer at a concentration of 10 μM, and 8 μL of molecular-grade water. Reactions were carried out using a MultiGene OptiMax thermal cycler (Labnet International, Edison, NJ, USA). Negative controls, in which genomic DNA was omitted from the reaction, were included to monitor for potential contamination. Thermal cycler protocols for each gene segment were as follows (primer names indicate the base-pair range where the primers bind along the transcript):

(I) ts18SF/18S650R: 95 °C initial heating for 5 min, followed by 28–30 cycles at 95 °C for 30 s, 62.7 °C for 30 s, and 72 °C for 50 s, ending with a 10 min extension at 72 °C;
(II) 18S450F/18S1500R: 95 °C initial heating for 5 min, followed by 28–30 cycles at 95 °C for 30 s, 62.4 °C for 30 s, 72 °C for 1 min and 10 s ending with a 10 min extension at 72 °C;
(III) 18S1350F/18S2230R: 95 °C initial heating for 5 min, followed by 28–30 cycles at 95 °C for 30 s, 64.5 °C for 30 s, 72 °C for 1 min and 5 s, ending with a 10 min extension at 72 °C.

PCR products were prepared for sequencing using Exo-CIP Rapid PCR Cleanup (New England Biolabs, Ipswich, MA, USA) or Zymoclean™ Gel DNA Recovery Kit (Zymo Research, Irvine, CA, USA). Sequencing of purified PCR products was carried out at Eurofins Genomics (Louisville, KY, USA) using an industry-standard Sanger sequencing methodology. All sequences generated as a part of this study were submitted to GenBank (Table 1).

High-quality percentage scores (HQ%) (i.e., the number of bases exceeding a 95% confidence threshold divided by the total number of bases in the untrimmed sequence, multiplied by 100) were calculated from the raw, untrimmed chromatograms prior to sequence editing or alignment. Following this initial quality assessment, low-quality sequences at the 5′ and 3′ ends were trimmed, reverse reads were reverse-complemented, and forward and reverse reads were aligned. Homologous sequences from multiple individuals were examined manually for potential ambiguous or conflicting base calls using the chromatogram traces to distinguish between potential sequencing errors and reproducible nucleotide variation. Additionally, the 18S locus of 65 individuals examining 10 families and 11 genera (ranging from 1206–2220 base pairs) was examined to determine the number of ambiguities (i.e., heterozygosity) within individuals and intraspecific variability by examining the chromatograms and via MAFFT alignments (Table S1).

### 2.5. Distance Matrices and Phylogenetic Analysis

To assess the ability of the V4/V5 hypervariable region of the 18S rRNA gene to function as a potential barcoding gene for Amphipoda, the gene sequence region amplified by the 18S450F/18S1500R primer pair was analyzed for closely related species in three different families: Crangonyctidae (genus *Crangonyx* Bate, 1859), Gammaridae (genus *Gammarus* Fabricius, 1775), and Hyalellidae (genus *Hyalella* Smith, 1874). For species in the genera *Crangonyx* and *Hyalella*, sequences were either downloaded from GenBank or generated as part of this study (Table 1), aligned, and trimmed to the length of the 18S450F/18S1500R region.

For the genera *Crangonyx* and *Hyalella*, uncorrected *p*-distances were calculated from sequence alignments using the dist.dna function in the ape package (v. 5.8-1 [60]). Dendrograms and heatmaps of genetic distances were produced in R using the gplots package (v. 3.1.3.1, see https://github.com/talgalili/gplots accessed on 6 May 2026), with default settings under the heatmap.2 function. For the genus *Gammarus*, the sequences of two specimens morphologically identified as *Gammarus minus*, from Scherr Spring, West Virginia, USA, were compared with members of the clades within the species complex identified by Cannizzaro et al. [61] using IQ-TREE v2.6 [62] and utilizing the MODELFINDER algorithm to select the best-fitting substitution models, which were analyzed under an edge-linked model. The Three-Parameter Model with unequal base frequencies, empirical base frequencies, and a proportion of invariable sites (TPM3u+F+I) was used. Statistical support was estimated using 10,000 ultrafast bootstrap replicates [63] and the Shimodaira–Hasegawa approximate likelihood test [64,65].

### 2.6. Species Delimitation

Species delimitation was performed for the *Crangonyx* and *Hyalella* species for both the 18S rRNA locus, as well as the COI locus for comparison. Two models were used: Automatic Barcode Gap Discovery (ABGD) [66] and the Assemble Species by Automatic Partitioning (ASAP) [67] employing the Spart Explorer platform ([68]; https://spartexplorer.mnhn.fr/delimitation accessed on 2 July 2026 ). The Kimura 2-parameter model (K80) was employed, which allows for transitions and transversions to occur at different rates, thereby accounting for differences in substitution patterns, with a transition/transversion ratio of 2.0, and all other parameters set to default values (ABGD: X = 1.5, Pmin = 0.001, and Pmax = 0.1; number of steps in Pmin, Pmax = 10, and number of bins = 20; ASAP: split groups below a probability of 0.001, number of best scores = 10, and a fixed seed value = −1). The species delimitation model was not used for the *Gammarus minus* phylogeny, as the goal of this analysis was to determine whether the 18S rRNA locus alone could reconstruct the three phylogeographic clade model identified by Cannizzaro et al. [61] and to determine whether sequences generated for the two *G. minus* individuals collected from Scherr Spring, West Virginia, would correctly align phylogeographically.

## 3. Results

### 3.1. Amplification and Sequencing

All permutations of primer pair amplifications produced high-quality reproducible amplicons for all 17 individuals tested (Table 1), with most sequences exhibiting pretrimmed chromatogram High-Quality (HQ) scores > 80%. Mean HQ% scores and ranges for the 17 individuals tested were as follows: 18SF: mean = 91.2%, range = 84.0–95.1%; 18S650R: mean = 90.5%, range = 62.7–96.8%; 18S450F: mean = 86.4%, range = 75.7–92.3%; 18S1500R: mean = 89.4%, range = 75.8–95.6%; 18S1350F: mean = 85.6%, range = 74–93.1%; 18S2230R: mean = 90.2%, range = 86.1–97.4%. Out of the 96 sequences, only 10 were <80% HQ score. All primer pairs successfully amplified the target regions across all tested species, with approximately 92% of amplicons producing useable sequences on the initial attempt. Amplicons that did not successfully sequence on the first attempt produced useable sequences upon resequencing. All sequences were deposited in GenBank (Table 1 and Table S1).

The examination of 65 individuals to determine the number of ambiguities (i.e., heterozygosity) demonstrated zero heterozygous sequences. In addition, the maximum intraspecific base pair differences across the 23 species examined was one base pair (Table S1).

### 3.2. Phylogenetic Analyses

**Figure 2:**
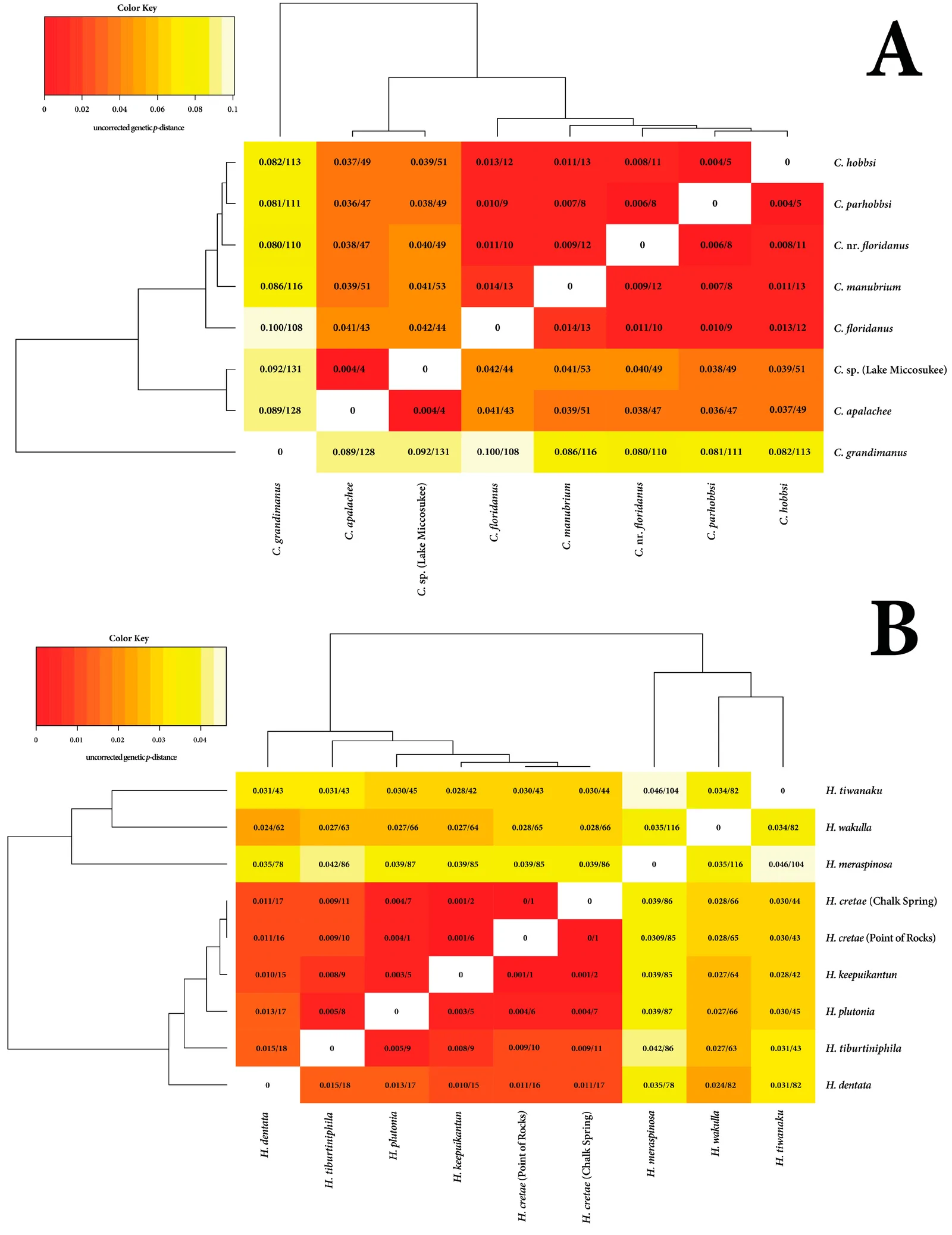
Genetic distance comparison (uncorrected genetic *p*-distance/base pairs) of the 18S rRNA among selected members of the genus *Crangonyx* (**A**) and *Hyalella* (**B**) based on the region of the 18S rRNA amplified by the 18S450F-18S1500R primers, which amplify the AROH.

**Figure 3:**
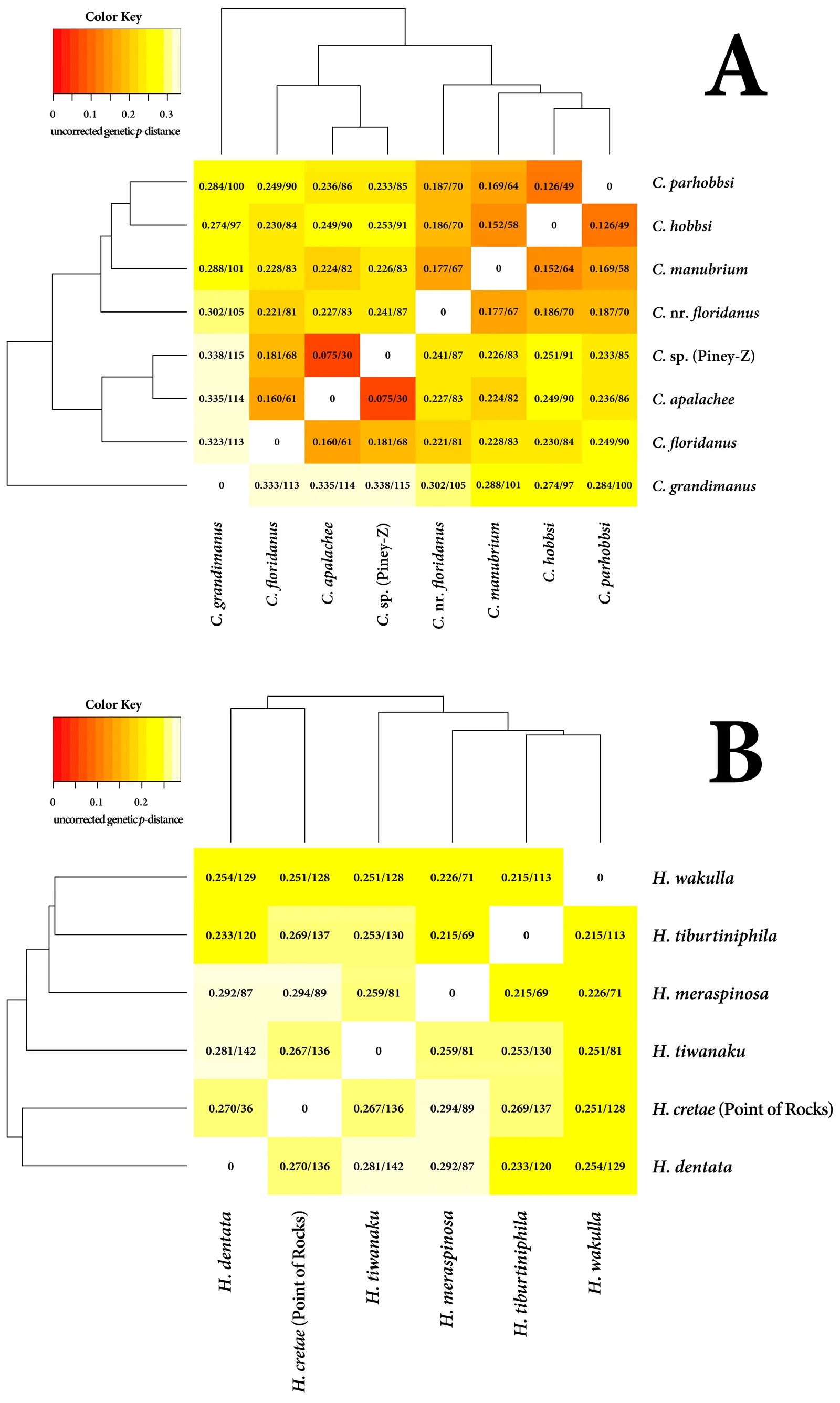
Genetic distance comparison (uncorrected genetic *p*-distance/base pairs) of COI among selected members of the genus *Crangonyx* (**A**) and *Hyalella* (**B**).

**Figure 4:**
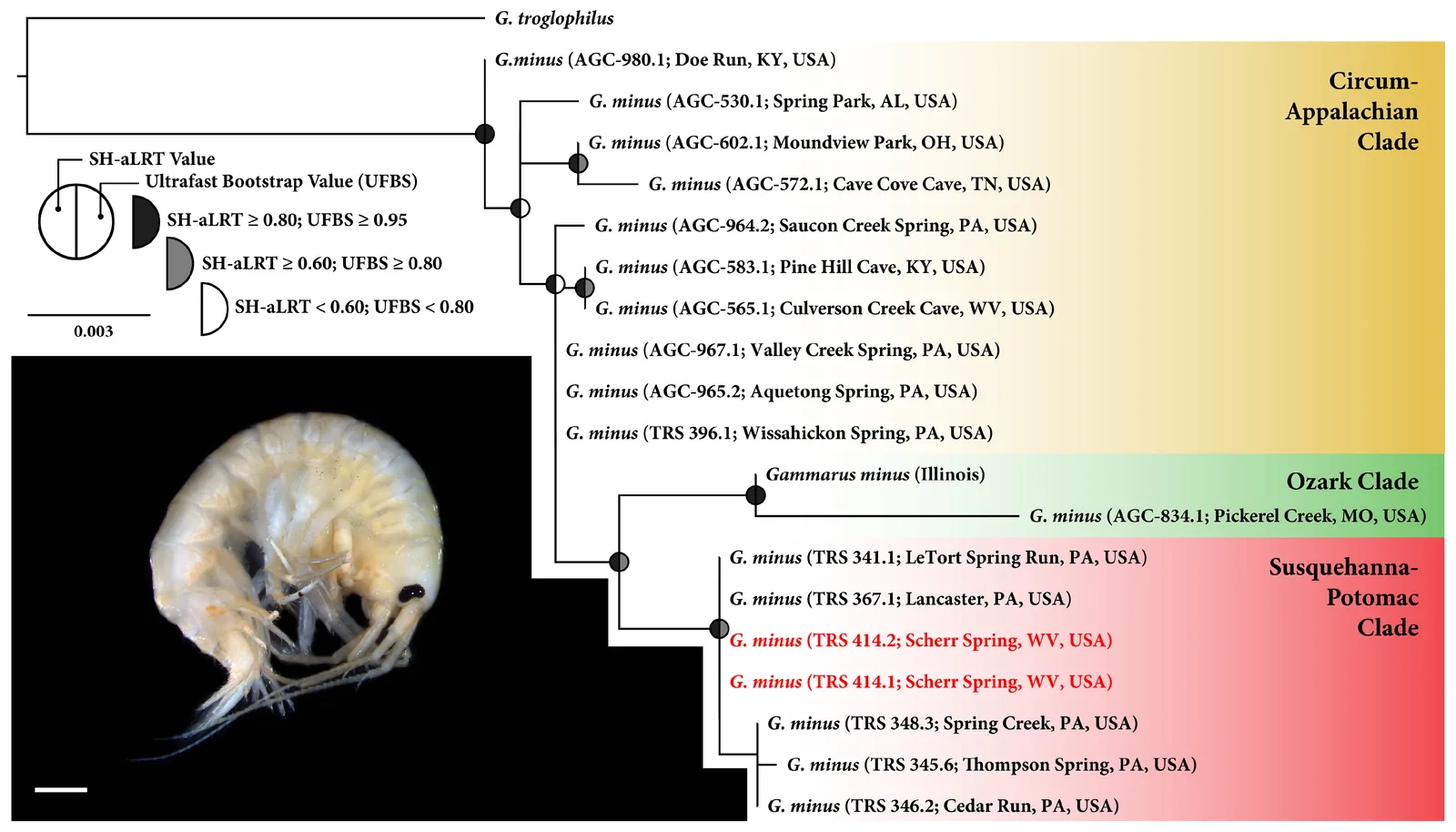
Maximum-likelihood phylogeny reconstructed for selected members of the genus *Gammarus* using IQ-TREE. Node support is indicated by shaded circles placed on the nodes; weakly supported nodes (<50%) are not marked. Lineages within *Gammarus minus*, as identified by Cannizzaro et al. [61], are marked by colored gradients, and newly sequenced individuals (Scherr Spring, WV, USA) are colored in red. Inset: *G. minus* Scherr Spring, West Virginia, USA; scale bar = 1 mm. Abbreviations: SH-aLRT, Shimodaira–Hasegawa approximate likelihood ratio test; UFBS, ultrafast bootstrap.

The dendrograms reconstructed for closely related *Crangonyx* and *Hyalella* species using only the region amplified by the 18S450F-18S1500R primers differentiated even closely related species (i.e., *C. hobbsi* Shoemaker, 1941/*C. parhobbsi* Cannizzaro and Sawicki, 2020; *H. plutonia* Cannizzaro, Lang & Berg, 2025/*H. keepuikantun* Cannizzaro, Lang & Berg, 2025) with *p*-distances ranging from 0.001–0.03 (Figure 2). The dendrogram reconstructing *Crangonyx* and *Hyalella* species using the COI locus clearly differentiated even closely related species with *p*-distances ranging from 0.075–0.338 (Figure 3). The maximum-likelihood tree examining the *Gammarus minus* metapopulation successfully reproduced the three clades defined by Cannizzaro et al. [61]; (I) the Susquehanna-Potomac clade containing populations within the Susquehanna drainage of central Pennsylvania and the Potomac drainage of West Virginia; (II) the Ozark clade containing populations from southern Illinois and Missouri; (III) the broadly distributed Circum-Appalachian clade ranging from eastern Pennsylvania, south down the Appalachian Mountains, and west into the interior plateau (Figure 4). The two individuals from Scherr Spring in West Virginia fell out in the Susquehanna-Potomac clade (Figure 4).

### 3.3. Species Delimitation

18S rRNA locus: for *Crangonyx* species, the ASAP analysis produced seven partitions, successfully differentiating all taxa as separate species, except *C. hobbsi* and *C. parhobbsi* (partition = 1; ASAP score = 2.5; probability = 1.237525 × 10^−1^); however, the ABGD analysis produced eight partitions, successfully differentiating *C. hobbsi* and *C. parhobbsi* as separate species (Table 5). For the *Hyalella* species the ASAP analysis produced four partitions (partition = 1; ASAP score = 1.5; probability = 4.091816 × 10^−1^); however, the ABGD analysis produced seven partitions, successfully differentiating all terminal taxa, except for *H. cretae* and *H. keepuikantun*, which exhibited a *p*-distance of 0.001 (Table 5). COI locus: both the ASAP and ABGD models successfully partitioned all examined species in both genera; however, for *Hyalella*, only six of the eight species (and nine individuals) analyzed for the 18S locus were analyzed for COI, as the *H. cretae* (Chalk Spring) individual, *H. keepuikantun*, and *H. plutonia* only produced pseudogenes (as indicated by multiple stop codons across all three reading frames when analyzed as protein coding mitochondrial DNA) and were, therefore, excluded from the analysis (Table 5).

## 4. Discussion

Species delimitation has long been dominated by highly variable and rapidly evolving mitochondrial loci such as COI, with nuclear loci such as the 18S rRNA often being characterized as too conserved to be used for species delimitation [69,70]. Schram and Koenemann [71] considered the 18S rRNA gene as only having an ‘appropriate level of use’ at the high-level ranks of phylum, class, and order for crustaceans. While much of the 18S rRNA locus is highly conserved, several ‘hypervariable’ regions are present within the locus, which are considerably less conserved [33]. These hypervariable regions have been successfully used as a target for species delimitation in a variety of contexts, including environmental DNA analysis [72] and barcoding [38,73]. Relative to amphipods, significant work has been conducted to show the utility of the locus for species delimitation across multiple families, including leucothoids [41,42], parabogidiellids and crangonyctids [43,44], and gammarids [12,16].

Given that most peracarid crustaceans have a unique insertion in the V4 hypervariable region and amphipods are unique amongst the peracarids with an additional insertion in the V5 hypervariable region (Figure 1; Ref. [33]), amphipods are especially poised to benefit from using the 18S to supplement COI. The primer pair 18S450F/1500R targets the AROH, and, thus, this region of the 18S could be useful for barcoding amphipod crustaceans. Here, we tested this utility using three different amphipod families. These analyses illustrate the potential of this region to distinguish among even closely related species and even phylogenetic variation within species complexes, with all of the species considered here displaying some level of differentiation (Figure 2 and Figure 3).

*Crangonyx floridanus* Bousfield, 1963, was thought to be native to and broadly distributed across the eastern United States, and it was subsequently introduced to various regions in the western United States, Japan, England, and Ireland and is considered invasive in Japan [18,74]. *Crangonyx floridanus* was first recorded in Japan by Tamura [75]. John Holsinger, a leading authority on crangonyctid taxonomy, morphologically identified and thoroughly described these amphipods as *C. floridanus* (Morino et al. [76]). However, Sawicki et al. [18] used the 18S V4 hypervariable region to show that the introduced populations in Japan (as well as in western United States, England, and Ireland) were not *C. floridanus* sensu stricto were but phylogenetically aligned with populations identified as *C*. nr. *floridanus* from Ohio, Pennsylvania, and northern Alabama.

Previous research conducted on the *C. floridanus* complex, as well as closely related *Crangonyx hobbsi* and *C. parhobbsi* species, demonstrated significant issues in amplifying and sequencing COI [18,28,77]. Thus, the *C. floridanus* complex provides a compelling use case in which an ecologist or environmental scientist collects a putative *C. floridanus* sample that could represent a possible new range extension but has difficulty amplifying the COI. In such circumstances, the AROH would provide an excellent target for identification, not only for verifying the population as *C. floridanus* sensu lato but also for resolving its identity to the specific level within the complex.

Relative to the 18S locus, the ASAP species delimitation model did not partition the closely related congeners *Crangonyx hobbsi* and *C. parhobbsi* and only produced four partitions for *Hyalella* spp.; however, the ABGD model fully partitioned all eight *Crangonyx* spp. and partitioned seven of the eight *Hyalella* spp. (Table 5). Both the ASAP and ABGD models were able to successfully partition all species analyzed using the more variable COI locus. However, three *Hyalella* species were not included in the COI analyses (*H. cretae* from Chalk Spring; *H. keepuikantun*; and *H. plutonia*) due to the amplification and sequencing of pseudogenes. Single locus (or even multiple loci) approaches can produce different partitions using different models. This is particularly true amongst closely related congeners with shallow divergences [78]. The fact that ABGD was able to recover all but one pair of the tested nominal species at the 18S locus demonstrates that the AROH contains species-level information, although its resolving power depends on the degree of lineage divergence and the methodology used. For instance, ASAP did not partition congeners exhibiting uncorrected *p*-distances of 0.001. However, whether or not the AROH can be used to consistently, repeatedly, and accurately partition closely related congeners does not invalidate its potential use for species identification. The 18S locus has often been considered too conserved to be of any use for species-level phylogenetics [69,70,71], but this analysis demonstrates that the AROH does have species resolving power. Importantly, even if a given species delimitation model lumps species together (as ASAP did for species exhibiting an uncorrected *p*-distance of 0.001), a 0.001 *p*-distance is sufficient to accurately differentiate unknown individuals to the correct species when conducting BLAST searches in databases such as GenBank. In other words, even if a species delimitation model fails to accurately partition, the differentiation can still be sufficient to barcode.

Although the cytochrome oxidase I (COI) gene is commonly used for barcoding [79], NUMTs [25,26,27,28,77,80,81,82], amplification and sequencing of bacterial genes when using common universal COI primers such as the Folmer [29] LCO1490/HCO2198 primers [17,30,31], and difficulty amplifying the COI, which may require the design of custom internal primers [17,22], can periodically make working with the locus difficult. Interestingly, three of the *Hyalella* species analyzed here produced pseudogenes, including *H. cretae* from Chalk Spring, although *H. cretae* from Point of Rocks was successfully sequenced, demonstrating intraspecific sequencing variability at this locus. Thus, the 18S rRNA gene has significant potential utility for barcoding amphipods, particularly when the COI locus does not easily amplify. The primers are highly conserved and, as a nuclear gene possesses no mitochondrial/microbial analogues, ensure that the amplification and sequencing of the correct target region are likely. The variability and length of the V4/V5 hypervariable region provide a target that should generally be useful for distinguishing among closely related species and even allow for phylogeographic delineation among broadly distributed species (Figure 2 and Figure 3, and Figure S1; Refs. [18,61]).

Although 18S rRNA is part of a multicopy ribosomal gene array (Libertini and Rampin [83]), no heterozygous sites or evidence of intragenomic sequence polymorphism was detected among the 65 amphipod individuals representing 10 families and 11 genera examined in this study (Table S1). This trend has been demonstrated across other amphipod taxa. For instance, Beermann et al. [84] found no intragenomic or intraspecific variation among 18S rRNA sequences of *Epimeria cornigera* (Fabricius, 1779) and *E. frankei* (Beerman & Raupach, 2018) in Beerman et al. (2018), which they attributed to “concerted evolution of the rRNA gene clusters”. Their findings are consistent with the sequence homogeneity observed in the present study, providing further evidence for strong evolutionary pressure to maintain sequence homogeneity across 18S copies in amphipods.

*Hyalella cretae* (Cannizzaro, Lange & Berg, 2023) was described from two locations, Chalk Spring and Point of Rocks Springs (both in Ash Meadows National Wildlife Refuge, Nye County, Nevada), and the populations from these two locations are identical across the AROH, although they demonstrate moderate divergence across the more variable mitochondrial 16S rRNA gene (Figure 2; Ref. [81]). These data suggest that the 18S may not always be variable enough to differentiate across subpopulations, and possibly subspecies, but also provides additional support for the low occurrence of heterozygosity.

However, when intraspecific variation is significant enough, the 18S locus can accurately partition populations phylogeographically (Figure 4). Cannizzaro et al. [61] conducted a phylogeographic study of the *G. minus* metapopulation. Their results demonstrated that three clades exist within this broadly distributed species: (I) the Susquehanna-Potomac clade containing populations within the Susquehanna drainage of central Pennsylvania and the Potomac drainage of West Virginia; (II) the Ozark clade containing populations from southern Illinois and Missouri; and (III) a broadly distributed Circum-Appalachian clade ranging from eastern Pennsylvania, south down the Appalachian Mountains, and west into the interior plateau. We recently received specimens of *G. minus* from Scherr Spring, in Grant County, West Virginia, which is located within the Potomac River drainage basin (Table 1). Our results demonstrate that the AROH alone can accurately reproduce the results of the multilocus analysis conducted by Cannizzaro et al. [61], accurately reproducing all three clades (Figure 4), in addition the Scherr Spring, West Virginia, populations nested within the Susquehanna-Potomac clade, as would be predicted by their biogeography (Figure 4).

When compared with the 18S rRNA, COI, as a commonly used barcoding gene, enjoys the advantage of 25+ years of data accumulation, with considerable amounts of sequence data on publicly available databases such as GenBank [85]. For instance, the 18S rRNA sequences produced during this study for the species *Cymadusa compta* (Smith in Verrill & Smith, 1874) and *Apocorphium lacustre* (Vanhöffen, 1911) are the first uploaded to GenBank for these species, although multiple COI sequences for both species are found in the database. This shortfall in data can be rectified by the incorporation of the full 18S rRNA gene (or at least enough of the gene to capture the AROH) as part of new species descriptions and phylogenetic analyses. To that end, we used 17 different species representing two suborders and 10 families for primer validation and analyses in this study, and we have subsequently verified the efficacy of these primers across scores of species that will be incorporated into future studies.

## 5. Conclusions

For Amphipoda, the 18S rRNA may be able to perform with more utility in barcoding and species-level phylogenetics than is traditionally thought. Although we are not suggesting 18S should be used as a substitute for COI, 18S can supplement COI, particularly if difficulty in amplification and sequencing of the COI gene occurs. With both highly conserved and variable regions, the 18S rRNA locus can help stabilize phylogenies, particularly when deeply rooted taxa are used, and simultaneously complement more variable genes for the phylogenetic partitioning of closely related species. Its inclusion as a staple locus in the examination of amphipod phylogenetics will not only accelerate the amount of data available for barcoding use but also greatly complement other loci while generating useful data.

## Figures and Tables

**Table 1:** Individuals that were used for various aspects of this study. Species that were amplified to test the new 18S rRNA primers (shaded light-gray). Unshaded cells are species used to create the dendrograms and phylogenetic tree. GenBank accession numbers for individuals collected and sequenced as part of this study are in bold.

Suborder	Family	Species	Location	Accession #
Senticaudata	Ampithoidae	*Cymadusa compta*	Kiwanis Park, Brevard County, FL	**QB063750** **.1**
	Corophiidae	*Apocorophium lacustre*	Shell Harbor, Putnam County, FL	**QB063755** **.1**
	Crangonyctidae	*Crangonyx grandimanus*	Indian Cave, Marion County, FL	PQ522380
		*Stygobromus floridanus*	Hole in the Wall Cave, Jackson County, FL	**QB063747** **.1**
		*Crangonyx apalachee*	Lake Jackson, Leon County, FL	OM845753
		*Crangonyx* sp.	Lake Miccosukee, Jefferson County, FL	OM845750
		*Crangonyx hobbsi*	Herzog Cave, Alachua County, FL	PQ522382
		*Crangonyx parhobbsi*	Madison Blue Spring, Madison County, FL	PQ522379
		*Crangonyx manubrium*	Hole in the Wall Cave, Jackson County, FL	PQ522353
		*Crangonyx* nr. *floridanus*	LeTort Spring Run, Cumberland County, PA	PQ522385
		*Crangonyx floridanus sensu stricto*	Cross Creek, Alachua County, FL	AJ966707
	Gammaridae	*Gammarus minus*	Scherr Spring, Grant County, WV	**QB063753**
		*Gammarus minus*	Scherr Spring, Grant County, WV	**QB063754**
		*Gammarus minus*	Spring Creek, Warren County, PA	**QB063757**
		*Gammarus minus*	Thompson Spring, Centre County, PA	**QB063758**
		*Gammarus minus*	Cedar Run, Lycoming County, PA	PZ274481
		*Gammarus minus*	Conestoga River Tributary, Lancaster County, PA	PZ274484
		*Gammarus minus*	LeTort Spring Run, Cumberland County, PA	PZ274479
		*Gammarus minus*	Pickerel Creek, Greene County, MO	PZ274456
		*Gammarus minus*	Wissahickon Spring, Philadelphia County, PA	**QB063761**
		*Gammarus minus*	Aquetong Spring, Bucks County, PA	PZ274470
		*Gammarus minus*	Valley Creek Spring, Chester County, PA	PZ274473
		*Gammarus minus*	Culverson Creek Cave, Greenbriar County, WV	PZ274461
		*Gammarus minus*	Pine Hill Cave, Rockcastle, KY	PZ274463
		*Gammarus minus*	Saucon Creek Spring, Lehigh County, PA	PZ274468
		*Gammarus minus*	Cave Cove Cave, Franklin County, TN	PZ274462
		*Gammarus minus*	Moundview Park, Hamilton County, OH	PZ274464
		*Gammarus minus*	Spring Park, Colbert County, AL	PZ274458
		*Gammarus minus*	Doe Run, Meade County, KY	PZ274475
		*Gammarus minus*	Illinois	EF582918
		*Gammarus troglophilus*	Rich’s Cave, Union County, IL	PQ814315
		*Gammarus lecroyae*	Wakulla River, Wakulla County, FL	**QB063759**
	Artesiidae	*Artesia subterranea*	Comal Springs, Comal County, TX	**QB063743**
	Hadziidae	*Paraholsingerius smaragdinus*	Dolan Spring, Val Verde County, TX	**QB063741**
	Hyalellidae	*Hyalella wakulla*	Wakulla River, Wakulla County, FL	**QB063760**
		*Hyalella cretae*	Chalk Spring, Nye County, NV	OR763358
		*Hyalella cretae*	Point of Rocks, Nye County, NV	OR763363
		*Hyalella tiwanaku*	Lago Umayo, Atuncolla District, Peru	MT823224
		*Hyalella meraspinosa*	Ash Spring, Lincoln County, NV	**QB075619**
		*Hyalella dentata*	Aspen Lake, El Paso county, CO	PX093675
		*Hyalella tiburtiniphila*	Hot Creek Spring, Nye County, NV	**QB075620**
		*Hyalella plutonia*	spring near Hwy 95/Oleo Rd, Nye County, NV	PX560105
		*Hyalella keepuikantun*	Devils Hole II, Nye County, NV	PX560103
	Talitridae	*Mexorchestia carpenteri*	Wakulla Beach, Wakulla County, FL	**QB063739**
	Brevitalitridae	*Talitroides alluaudi*	Ocala Rd, Leon County, FL	**QB063740**
Amphilochidea	Seborgiidae	*Seborgia relicta*	Comal Springs, Comal County, TX	**QB063742**

**Table 2:** GenBank accession numbers used to design the 18S rRNA primers in this study.

Suborder	Family	Species	Accession #
Amphilochidea	Amphilochidae	*Amphilochius tenuimanus*	DQ378029
	Eusiridae	*Eusirus* cf. *microps*	DQ378011
	Haustoriidae	*Haustorius arenarius*	AY826950
	Vicmusiidae	*Acanthonotozomopisis pushkini*	DQ378038
	Iphimediidae	*Pariphimedia integricauda*	DQ378001
	Leucothoidae	*Leucothoe spinicarpa*	DQ378025
	Tryphosidae	*Lepidepecreum umbo*	AY826968
	Uristidae	*Onisimus glacialis*	JF266614
		*Uristes murrayi*	AY826965
	Oedicerotidae	*Bathymedon obtusifrons*	AF419236
		*Monoculodes packardi*	DQ378015
		*Paroediceros propinquus*	AF419231
Colomastigidea	Colomastigidae	*Colomastix fissilingua*	DQ378032
Hyperiidea	Hyperiidae	*Cyllopus lucasii*	DQ378042
		*Hyperia galba*	DQ378046
		*Themisto compressa*	DQ378048
	Mimonectidae	*Mimonectes loveni*	KC428907
	Vibiliidae	*Vibilia antarctica*	DQ378044
Senticaudata	Corophiidae	*Corophium volutator*	DQ378027
	Crangonyctidae	*Uralocrangonyx chlebnikovi*	HQ286017
		*Synurella ambulans*	HQ286018
	Gammaridae	*Gammarus wilkitzkii*	JF266609
	Hyalidae	*Parhyale hawaiensis*	AY826957
	Talitridae	*Deshayesorchestia deshayesi*	AY826956
Ingolfiellida	Ingolfiellidae	*Ingolfiella tabularis*	DQ378054

**Table 3:** Primer names and sequences designed for this study along with melting temperatures and GC% for each primer. Primer names indicate the base pair range where the primers bind along the transcript.

Primer Name	Primer Sequences 5′-3′	*T*m (°C)	GC%
ts18SF	GATCCTGCCAGTAGTCATATGC	62.7	50.0
18S650R	GAATTACCGCGGCTGCTGGC	66.6	65.0
18S450F	GATTCCGGAGAGGCAGCC	64.5	66.7
18S1500R	GGTGCCTTTCCGTCAATTCC	62.4	55.0
18S1350F	CGAAGGCGATCAGATACCGC	64.5	60.0
18S2230R	CAATCGGTAGTAGCGACGGGC	66.5	61.9

**Table 4:** Primer sequence conformity across all species used for the primer design. Dots indicate no base pair differences. Dashes in the sequences of *Parhyale hawaiensis* (Dana, 1853), *Deshayesorchestia deshayesi* (Audouin, 1826) (ts18SF), and *Acanthonotozomopsis pushkini* (Bushueva, 1978) (18S2230R) indicate insertions. The primer 18S1350F exhibited 100% sequence conformity across all species and, thus, is not listed. All sequences are written in a 5′–3′ direction.

Species	Sequence Conformity				
	ts18SF GATCCTGCCAGTAGTCATATGC	18S650R GAATTACCGCGGCTGCTGGC	18S450F GATTCCGGAGAGGCAGCC	18S1500R GGTGCCTTTCCGTCAATTCC	18S2230R CAATCGGTAGTAGCGACGGGC
*Amphilochius tenuimanus*	· · · · · · · · · · · · · · · T· · · · · ·	· · · · · · · · · · · · · · · · · · · ·	· · · · · · · · · · · · · · · · · ·	· · · · · · · · · · · · · · · · · · · ·	· · · · · · · · · · · · · · · · · · · · ·
*Eusirus* cf. *microps*	· · · · · · · · · · · · · · · · · · · · · ·	· · · · · · · · · · · · · · · · · · · ·	· · · · · · · · · · · · · · · · · ·	· · · · · · · · · · · · · · · · · · · ·	· · · · · · · · · · · · · · · · · · · · ·
*Haustorius arenarius*	· · · · · · · · · · · · · · · · · · · · · ·	· · · · · · · · · · · · · · · C· · · ·	· · · · · · · · · · G· · · · · · ·	· · · · · · · · · · · · · · · · · · · ·	· · · · · · · · · · · · · · · · · · · · ·
*Acanthonotozomopsis pushkini*	· · · · · · · · · · · · · · · · · · · · · ·	· · · · · · · · · · · · · · · · · · · ·	· · · · · · · · · · · · · · · · · ·	· · · · · · · · · · · · · · · · · · · ·	· · · · · · · · · · · T· **^–^** · · · · · · ·
*Pariphimedia integricauda*	· · · · · · · · · · · · · · · · · · · · · ·	· · · · · · · · · · · · · · · · · · · ·	· · · · · · · · · · · · · · · · · ·	· · · · · · · · · · · · · · · · · · · ·	· · · · · · · · · · · · · · · · · · · · ·
*Leucothoe spinicarpa*	· · · · · · · · · · · · · · · · · · · · · ·	· · · · · · · · · · · · · · · · · · · ·	· · · · · · · · · · · · · · · · · ·	· · · · · · · · · · · · · · · · · · · ·	· · · · · · · · · · · · · · · · · · · · ·
*Lepidepecreum umbo*	· · · · · · · · · · · · · · · · · · · · · ·	· · · · · · · · · · · · · · · · · · · ·	· · · · · · · · · · · · · · · · · ·	· · · · · · · · · · · · · · · · · · · ·	· · · · · · · · · · · · · · · · · · · · ·
*Onisimus glacialis*	· · · · · · · · · · · · · · · · · · · · · ·	· · · · · · · · · · · · · · · · · · · ·	· · · · · · · · · · · · · · · · · ·	· · · · · · · · · · · · · · · · · · · ·	· · · · · · · · · · · · · · · · · · · · ·
*Bathymedon obtusifrons*	· · · · · · · · · · · · · · · G· · · · · ·	· · · · · · · · · · · · · · · · · · · ·	· G· · · · · · · · · · · · · · · ·	· · · · · · · · · · · · · · · · · · · ·	· · · · · · · · · · · · · · · · · · · · ·
*Monoculodes packardi*	· · · · · · · · · · · · · · · G· · · · · ·	· · · · · · · · · · · · · · · · · · · ·	· G· · · · · · · · · · · · · · · ·	· · · · · · · · · · · · · · · · · · · ·	· · · · · · · · · · · · · · · · · · · · ·
*Colomastix fissilingua*	· · · · · · · · · · · · · · · T· · · · · ·	· · · · · · · · · · · · · · · · · · · ·	· · · · · · · · · · · · · · · · · ·	· · · · · · · · · · · · · · · · · · · ·	· · · · · · · · T· · · · · · · · · · · ·
*Cyllopus lucasi*	· · · · · · · · · · · · AGTC· · · · · ·	· · · · · · · · · · · · · · · · · · · ·	· · · · · · · · · · · · · · · · · ·	· · · · · · · · · · · · · · · · · · · ·	· · · · · · · · · · · · · · · · · · · · ·
*Hyperia galba*	· · · · · · · · · · · · AGTC· · · · · ·	· · · · · · · · · · · · · · · · · · · ·	· · · · · · · · · · · · · · · · · ·	· · · · · · · · · · · · · · · · · · · ·	· · · · · · · · · · · · · · · · · · · · ·
*Themisto compressa*	· · · · · · · · · · · · AGTC· · · · · ·	· · · · · · · · · · · · · · · · · · · ·	· · · · · · · · · · · · · · · · · ·	· · · · · · · · · · · · · · · · · · · ·	· · · · · · · · · · · · · · · · · · · · ·
*Mimonectes loveni*	N/A	· · · · · · · · · · · · · · · · · · · ·	· · · · · · · · · · · · · · · · · ·	· · · · · · · · · · · · · · · · · · · ·	· · · · · · · · · · · · · · · · · · · · ·
*Vibilia antarctica*	· · · · · · · · · · · · AGTC· · · · · ·	· · · · · · · · · · · · · · · · · · · ·	· · · · · · · · · · · · · · · · · ·	· · · · · · · · · · · · · · · · · · · ·	· · · · · · · · · · · · · · · · · · · · ·
*Corophium volutator*	· · · · · · · · · · · · · · · · · · · · · ·	· · · · · · · · · · · · · · · · · · · ·	· · · · · · · · · · · · · · · · · ·	· · · · · · · · · · · · · · · · · · · ·	· · · · · · · · · · · · · · · · · · · · ·
*Uralcrangonyx chlebnikovi*	· · · · · · · · · · · · · · · · · · · · · ·	· · · · · · · · · · · · · · · · · · · ·	· · · · · · · · · · · · · · · · · ·	· · · · · · · · · · · · · · · · · · · ·	· · · · · · · · · · · · · · · · · · · · ·
*Synurella ambulans*	· · · · · · · · · · · · · · · · · · · · · ·	· · · · · · · · · · · · · · · · · · · ·	· · · · · · · · · · · · · · · · · ·	· · · · · · · · · · · · · · · · · · · ·	· · · · · · · · · · · · · · · · · · · · ·
*Gammarus wilkitzkii*	· · · · · · · · · · · · · · · · · · · · · ·	· · · · · · · · · · · · · · · · · · · ·	· · · · · · · · · · · · · · · · · ·	· · · · · · · · · · · · · · · · · · · ·	· · · · · · · · · · · · · · · · · · · · ·
*Paroediceros propinquus*	· · · · · · · · · · · · · · · G· · · · · ·	· · · · · · · · · · · · · · · · · · · ·	· G· · · · · · · · · · · · · · · ·	· · · · · · · · · · · · · · · · · · · ·	· · · · · · · · · · · · · · · · · · · · ·
*Parhyale hawaiensis*	· · · · · · · · · · · · · · · **^–^** · · · · · ·	· · · · · · · · · · · · · · · · · · · ·	· · · · · · · · · · · · · · · · · ·	· · · · · · · · · · · · · · · · · · · ·	· · · · · · · · · · · · · · · · · · · · ·
*Deshayesorchestia* *deshayesi*	· · · · · · · · · · · · · · · **^–^** · · · · · ·	· · · · · · · · · · · · · · · · · · · ·	· · · · · · · · · · · · · · · · · ·	· · · · · · · · · · · · · · · · · · · ·	· · · · · · · · · · · · · · · · · · · · ·
*Uristes murrayi*	· · · · · · · · · · · · · · · · · · · · · ·	· · · · · · · · · · · · · · · · · · · ·	· G· · · · · · · · · · · · · · · ·	· · · · · · · · · · · · · · · · · · · ·	· · · · · · · · · · · · · · · · · · · · ·
*Ingolfiella tabularis*	· · · · · · · · · · · · · · · · · · · · · ·	· · · · · · · · · · · · · · · · · · · ·	· · · · · · · · · · · · G· · · · ·	· · · · · · · · · · · · · G· · · · · ·	· · · · · · · · · · · · · · · · · · · · ·

**Table 5:** Summary of ABGD and ASAP species delimitation results. The COI species-delimitation analyses for *Hyalella* included fewer taxa than the corresponding 18S analyses, because three individuals produced COI pseudogene sequences (*H. cretae* from Chalk Spring; *H. keepuikantun*; and *H. plutonia*) and were, therefore, excluded from the COI dataset.

Genus	Locus	Method	Model/Score Information	Delimited Subsets	Results
*Crangonyx*	18S	ABGD	K80; MinSlope = 1.5; barcode gap distances = 0.001, 0.00167, 0.00278, 0.00464, 0.00774, 0.0129, 0.0215, 0.03594	8, 8, 8, 3, 3, 2, 2, 2	Stable high-resolution solution of all 8 species used in the analysis in partitions 1–3; progressively broader lumping at higher distance thresholds.
*Crangonyx*	18S	ASAP	ASAP score = 2.5; probability = 0.1237525	7 with recursion; 6 without recursion	Best ASAP partition recovered 7 groups with recursion; *C. hobbsi* and *C. parhobbsi* were grouped together.
*Crangonyx*	COI	ABGD	K80; MinSlope = 1.5; barcode gap distances = 0.001, 0.00167, 0.00278, 0.00464, 0.00774, 0.0129, 0.0215, 0.0359, 0.05995	8, 8, 8, 8, 8, 8, 8, 8, 8	All 8 taxa were consistently recovered as separate groups.
*Crangonyx*	COI	ASAP	ASAP score = 1.00; probability = 0.0001399986	8	Best ASAP partition recovered 8 groups.
*Hyalella*	18S	ABGD	K80; MinSlope = 1.5; barcode gap distances = 0.001, 0.00167, 0.00278, 0.00464, 0.007742637	7, 7, 7, 4, 4	Stable high-resolution solution of 7 of 8 species in partitions 1–3 (failing to resolve *H. cretae* and *H. keepuikantun*, which exhibited a *p*-distance of 0.001); broader 4-group solution in partitions 4–5.
*Hyalella*	18S	ASAP	ASAP score = 1.5; probability = 0.4091816	4 with recursion; 3 without recursion	Best ASAP partition recovered 4 groups with recursion; several Mojave/Nevada Hyalella lineages were grouped together.
*Hyalella*	COI	ABGD	K80; MinSlope = 1.5; barcode gap distances = 0.001, 0.00167, 0.00278, 0.00464, 0.00774, 0.0129, 0.0215, 0.0359, 0.05995	6, 6, 6, 6, 6, 6, 6, 6, 6	All 6 taxa were consistently recovered as separate groups.
*Hyalella*	COI	ASAP	ASAP score = 1.50; probability = 0.8862275	6	Best ASAP partition recovered 6 groups.

## Data Availability

Data underlying this article will be submitted to the GenBank Nucleotide Database at www.ncbi.nlm.nih.gov/genbank/ and can be accessed via the accession numbers provided in Table 1.

## Supplementary Materials

The following supporting information can be downloaded at: https://www.mdpi.com/article/10.3390/biology15191752/s1, Figure S1: Example alignment of the 18S AROH based on selected members of the *Crangonyx floridanus* species complex; Table S1: Comparison of the 18S locus across multiple species to determine the degree of ambiguities (i.e., heterozygosity) within individual sequences and the degree of intraspecific variability. Sequences highlighted bold were produced as part of this study..

## References

[1] Lowry J.K., Myers A.A. (2017). A phylogeny and classification of the Amphipoda with the establishment of the new order Ingolfiellida (Crustacea: Peracarida). Zootaxa.

[2] Ritter C.J., Bourne D.G. (2024). Marine amphipods as integral members of global ocean ecosystems. J. Exp. Mar. Biol. Ecol..

[3] Horton T., Lowry J., De Broyer C., Bellan-Santini D., Copilaş-Ciocianu D., Corbari L., Costello M.J., Daneliya M., Dauvin J.-C., Fišer C. World Amphipoda Database. https://www.marinespecies.org/amphipoda.

[4] Bousfield E.L. (1973). Shallow-water gammaridean Amphipoda of New England. Smithson. Contrib. Zool..

[5] LeCroy S.E. (2000). Volume 1: Families Gammaridae, Hadziidae, Isaeidae, Melitidae, and Oedicerotidae. An Illustrated Identification Guide to the Nearshore Marine and Estuarine Gammaridean Amphipoda of Florida.

[6] Jamieson A.J., Brooks L.S., Reid W.D., Piertney S.B., Narayanaswamy B.E., Linley T.D. (2019). Microplastics and synthetic particles ingested by deep-sea amphipods in six of the deepest marine ecosystems on Earth. R Soc. Open Sci..

[7] Sket B. (1999). The nature of biodiversity in hypogean waters and how it is endangered. Biodivers. Conserv.

[8] Väinölä R., Witt J.D.S., Grabowski M., Bradbury J.H., Jazdzewski K., Sket B. (2008). Global diversity of amphipods (Amphipoda; Crustacea) in freshwater. Hydrobiologia.

[9] Culver D.C., Pipan T. (2009). The Biology of Caves and Other Subterranean Habitats.

[10] Wellborn G.A., Witt J.D., Cothran R.D. (2015). Class Malacostraca, superorders Peracarida and Syncarida. Thorp and Covich’s Freshwater Invertebrates.

[11] Wattier R., Mamos T., Copilas-Ciocianu D., Jelic M., Ollivier A., Chaumot A., Danger M., Felten V., Piscart C., Zganec K. (2020). Continental-scale patterns of hyper-cryptic diversity within the freshwater model taxon *Gammarus fossarum* (Crustacea, Amphipoda). Sci. Rep..

[12] Smith L., Long R.A., Cannizzaro A.G., Sawicki T.R. (2024). Vicariance and cryptic diversity revealed by molecular phylogenetic analyses of estuarine *Gammarus* species (Crustacea: Amphipoda) due to formation of the Labrador Current. Invertebr. Syst..

[13] Delić T., Trontelj P., Rendoš M., Fišer C. (2017). The importance of naming cryptic species and the conservation of endemic subterranean amphipods. Sci. Rep..

[14] Hupało K., Teixeira M.A.L., Rewicz T., Sezgin M., Iannilli V., Karaman G.S., Grabowski M., Costa F.O. (2019). Persistence of phylogeographic footprints helps to understand cryptic diversity detected in two marine amphipods widespread in the Mediterranean basin. Mol. Phylogenet. Evol..

[15] Niemiller M.L., Cannizzaro A.G., Sawicki T.R., Culver D.C. (2024). A new species of *Stygobromus* Cope, 1872 (Amphipoda, Crangonyctidae) from a hypotelminorheic seepage spring in Washington, D.C., USA. Subterr. Biol..

[16] Coppock N.H., Cannizzaro A.G., Grant K.D., Sawicki T.R. (2026). The Florida Peninsula as an engine of estuarine evolution: Insights from *Gammarus daiberi* Bousfield, 1969 (Crustacea: Amphipoda). J. Biogeogr..

[17] Cannizzaro A.G., Lieb D.A., Çelikbaş B., Sawicki T.R. (2026). The Big Striped Beast: An Unexpected New Species of Freshwater Gammarus (Amphipoda: Gammaridae) from Cumberland County, Pennsylvania.

[18] Sawicki T.R., Cannizzaro A.G., Dowd S.E. (2025). Florida man exonerated: An amphipod case study on the implications of cryptic species complexes and global invasions. Biol. Invasions.

[19] Hebert P.D.N., Ratnasingham S., DeWaard J.R. (2003). Barcoding animal life: Cytochrome c oxidase subunit 1 divergences among closely related species. Proc. Biol. Sci..

[20] Waugh J. (2007). DNA barcoding in animal species: Progress, potential and pitfalls. BioEssays.

[21] Bucklin A., Steinke D., Blanco-Bercial L. (2011). DNA barcoding of marine metazoa. Annu. Rev. Mar. Sci..

[22] Kelly D.W., MacIsaac H.J., Heath D.D. (2006). Vicariance and dispersal effects on phylogeographic structure and speciation in a widespread estuarine invertebrate. Evolution.

[23] Ethridge J.Z., Gibson R.J., Nice C.C. (2013). Cryptic diversity within and amongst spring-associated *Stygobromus* amphipods (Amphipoda: Crangonyctidae). Zool. J. Linn. Soc..

[24] Gurkov A., Rivarola-Duarte L., Bedulina D., Fernández Casas I., Michael H., Drozdova P., Nazarova A., Govorukhina E., Timofeyev M., Stadler P.F. (2019). Indication of ongoing amphipod speciation in Lake Baikal by genetic structures within endemic species. BMC Evol. Biol..

[25] Hazkani-Covo E., Zeller R.M., Martin W. (2010). Molecular poltergeists: Mitochondrial DNA copies (numts) in sequenced nuclear genomes. PLoS Genet..

[26] Buhay J.E. (2009). COI-like sequences are becoming problematic in molecular systematic and DNA barcoding studies. J. Crustac. Biol..

[27] Schubart C.D., Martin J.W., Crandall K.A., Felder D.L. (2009). Mitochondrial DNA and decapod phylogenies: The importance of pseudogenes and primer optimization. Decapod Crustacean Phylogenetics.

[28] Cannizzaro A.G., Balding D., Lazo-Wasem E.A., Sawicki T.R. (2020). A new species rises from beneath Florida: Molecular phylogenetic analyses reveal cryptic diversity among the metapopulation of *Crangonyx hobbsi* Shoemaker, 1941 (Amphipoda: Crangonyctidae). Org. Divers Evol..

[29] Folmer O., Black M., Hoeh W., Lutz R., Vrijenhoek R. (1994). DNA primers for amplification of mitochondrial cytochrome c oxidase subunit I from diverse metazoan invertebrates. Mol. Mar. Biol. Biotechnol..

[30] Siddall M.E., Fontanella F.M., Watson S.C., Kvist S., Erseus C. (2009). Barcoding bamboozled by bacteria: Convergence to metazoan mitochondrial primer targets by marine microbes. Syst. Biol..

[31] Mioduchowska M., Czyż M.J., Gołdyn B., Kur J., Sell J. (2018). Instances of erroneous DNA barcoding of metazoan invertebrates: Are universal cox1 gene primers too ‘universal’?. PLoS ONE.

[32] Xie Q., Lin J., Qin Y., Zhou J., Bu W. (2011). Structural diversity of eukaryotic 18S rRNA and its impact on alignment and phylogenetic reconstruction. Protein Cell.

[33] Spears T., DeBry R.W., Abele L.G., Chodyla K. (2005). Peracarid monophyly and interordinal phylogeny inferred from nuclear small-subunit ribosomal DNA sequences (Crustacea: Malacostraca: Peracarida). Proc. Biol. Soc. Wash..

[34] Kakui K., Katoh T., Hiruta S.F., Kobayashi N., Kajihara H. (2011). Molecular systematics of Tanaidacea (Crustacea: Peracarida) based on 18S sequence data, with an amendment of suborder/superfamily-level classification. Zool. Sci..

[35] Abouheif E., Zardoya R., Meyer A. (1998). Limitations of metazoan 18S rRNA sequence data: Implications for reconstructing a phylogeny of the animal kingdom and inferring the reality of the Cambrian explosion. J. Mol. Evol..

[36] Adoutte A., Balavoine G., Lartillot N., Lespinet O., Prud’homme B., de Rosa R. (2000). The new animal phylogeny: Reliability and implications. Proc. Natl. Acad. Sci. USA.

[37] Van de Peer Y., Baldauf S.L., Doolittle W.F., Meyer A. (2000). An updated and comprehensive rRNA phylogeny of (crown) eukaryotes based on rate-calibrated evolutionary distances. J. Mol. Evol..

[38] Wu S., Xiong J., Yu Y. (2015). Taxonomic resolutions based on 18S rRNA genes: A case study of subclass Copepoda. PLoS ONE.

[39] Zhao Y., Zhang W.Y., Wang R.L., Niu D.L. (2020). Divergent domains of 28S ribosomal RNA gene: DNA barcodes for molecular classification and identification of mites. Parasit. Vectors.

[40] Chittavichai T., Sathitnaitham S., Utthiya S., Prompichai W., Prommarit K., Vuttipongchaikij S., Wonnapinij P. (2025). Limitations of 18S rDNA sequence in species-level classification of dictyostelids. Microorganisms.

[41] White K. (2011). A taxonomic review of the Leucothoidae (Crustacea: Amphipoda). Zootaxa.

[42] White K.N., Reimer J.D. (2012). DNA phylogeny of Ryukyus Leucothoidae (Crustacea: Amphipoda). Contrib. Zool..

[43] Cannizzaro A.G., Gibson J.R., Sawicki T.R. (2020). A new enigmatic genus of subterranean amphipod (Amphipoda: Bogidielloidea) from Terrell County, Texas, with the establishment of *Parabogidiellidae*, fam. nov., and notes on the family Bogidiellidae. Invertebr. Syst..

[44] Sisco J.M., Sawicki T.R. (2023). Molecular and morphological analyses reveal a new hypogean species of amphipod in the genus *Crangonyx* Bate, 1859 (Crustacea: Crangonyctidae) within the *floridanus* species complex, from Suwannee County, Florida. J. Nat. Hist..

[45] Machida R.J., Knowlton N. (2012). PCR primers for metazoan nuclear 18S and 28S ribosomal DNA sequences. PLoS ONE.

[46] Hadziavdic K., Lekang K., Lanzen A., Jonassen I., Thompson E.M., Troedsson C. (2014). Characterization of the 18S rRNA gene for designing universal eukaryote specific primers. PLoS ONE.

[47] Hugerth L.W., Muller E.E., Hu Y.O., Lebrun L.A., Roume H., Lundin D., Wilmes P., Andersson A.F. (2014). Systematic design of 18S rRNA gene primers for determining eukaryotic diversity in microbial consortia. PLoS ONE.

[48] Englisch U., Koenemann S. (2001). Preliminary phylogenetic analysis of selected subterranean amphipod crustaceans, using small subunit rDNA gene sequences. Org. Divers Evol..

[49] Best R.J., Stachowicz J.J. (2013). Phylogeny as a proxy for ecology in seagrass amphipods: Which traits are most conserved?. PLoS ONE.

[50] Chevaldonné P., Boris S., Marschal C., Lejeusne C., Calado R. (2008). Improvements to the Sket bottle: A simple manual device for sampling small crustaceans from marine caves and other cryptic habitats. J. Crustac. Biol..

[51] Bousfield E.L. (1969). New records of *Gammarus* (Crustacea: Amphipoda) from the Middle Atlantic region. Chesap. Sci..

[52] LeCroy S.E. (2002). Volume 2: Families Ampeliscidae, Amphilochidae, Ampithoidae, Aoridae, Argissidae, and Haustoriidae. An Illustrated Identification Guide to the Nearshore Marine and Estuarine Gammaridean Amphipoda of Florida.

[53] LeCroy S.E. (2004). Volume 3: Families Bateidae, Biancolinidae, Cheluridae, Colomastigidae, Corophiidae, Cyproideidae, and Dexaminidae. An Illustrated Identification Guide to the Nearshore Marine and Estuarine Gammaridean Amphipoda of Florida.

[54] Katoh K., Misawa K., Kuma K., Miyata T. (2002). MAFFT: A novel method for rapid multiple sequence alignment based on fast Fourier transform. Nucleic Acids Res..

[55] Katoh K., Standley D.M. (2013). MAFFT multiple sequence alignment software version 7: Improvements in performance and usability. Mol. Biol. Evol..

[56] Kearse M., Moir R., Wilson A., Stones-Havas S., Cheung M., Sturrock S., Buxton S., Cooper A., Markowitz S., Duran C. (2012). Geneious Basic: An integrated and extendable desktop software platform for the organization and analysis of sequence data. Bioinformatics.

[57] Rozen S., Skaletsky H. (2000). Primer3 on the WWW for general users and for biologist programmers. Bioinformatics Methods and Protocols.

[58] Kibbe W.A. (2007). OligoCalc: An online oligonucleotide properties calculator. Nucleic Acids Res..

[59] Chuvochina M., Gerken J., Frentrup M., Sandikci Y., Goldmann R., Freese H.M., Göker M., Sikorski J., Yarza P., Quast C. (2026). SILVA in 2026: A global core biodata resource for rRNA within the DSMZ digital diversity. Nucleic Acids Res..

[60] Paradis E., Schliep K. (2019). Ape 5.0: An environment for modern phylogenetics and evolutionary analyses in R. Bioinformatics.

[61] Cannizzaro A.G., Sawicki T.R., Lieb D.A., Berg D.J. (2026). Secrets of the spring-keepers: Phylogeographic analyses of a freshwater amphipod uncover dispersal pathways in eastern North America. J. Biogeogr..

[62] Minh B.Q., Schmidt H.A., Chernomor O., Schrempf D., Woodhams M.D., von Haeseler A., Lanfear R. (2020). IQ-TREE 2: New models and efficient methods for phylogenetic inference in the genomic era. Mol. Biol. Evol..

[63] Minh B.Q., Nguyen M.A.T., von Haeseler A. (2013). Ultrafast approximation for phylogenetic bootstrap. Mol. Biol. Evol..

[64] Shimodaira H., Hasegawa M. (1999). Multiple comparisons of log-likelihoods with applications to phylogenetic inference. Mol. Biol. Evol..

[65] Guindon S., Dufayard J.F., Lefort V., Anisimova M., Hordijk W., Gascuel O. (2010). New algorithms and methods to estimate maximum-likelihood phylogenies: Assessing the performance of PhyML 3.0. Syst. Biol..

[66] Puillandre N., Lambert A., Brouillet S., Achaz G. (2012). ABGD, Automatic Barcode Gap Discovery for primary species delimitation. Mol. Ecol..

[67] Puillandre N., Brouillet S., Achaz G. (2021). ASAP: Assemble species by automatic partitioning. Mol. Ecol. Resour..

[68] Miralles A., Ducasse J., Brouillet S., Flouri T., Fujisawa T., Kapli P., Knowles L.L., Kumari S., Stamatakis A., Sukumaran J. (2022). SPART: A versatile and standardized data exchange format for species partition information. Mol. Ecol. Resour..

[69] Tang C.Q., Leasi F., Obertegger U., Kieneke A., Barraclough T.G., Fontaneto D. (2012). The widely used small subunit 18S rDNA molecule greatly underestimates true diversity in biodiversity surveys of the meiofauna. Proc. Natl. Acad. Sci. USA.

[70] Vierna J., Cuperus J., Martinez-Lage A., Jansen J.M., Perina A., Van Pelt H., Gonzalez-Tizon A.M. (2014). Species delimitation and DNA barcoding of Atlantic Ensis (Bivalvia, Pharidae). Zool. Scr..

[71] Schram F.R., Koenemann S. (2021). Evolution and Phylogeny of Pancrustacea: A Story of Scientific Method.

[72] de Jonge D.S., Merten V., Bayer T., Puebla O., Reusch T.B., Hoving H.J.T. (2021). A novel metabarcoding primer pair for environmental DNA analysis of Cephalopoda (Mollusca) targeting the nuclear 18S rRNA region. R Soc. Open Sci..

[73] Zimmermann J., Jahn R., Gemeinholzer B. (2011). Barcoding diatoms: Evaluation of the V4 subregion on the 18S rRNA gene, including new primers and protocols. Org. Divers Evol..

[74] Zhang J., Holsinger J.R. (2003). Systematics of the freshwater amphipod genus *Crangonyx* (Crangonyctidae) in North America. Va. Mus. Nat. Hist. Mem..

[75] Tamura T. (1990). Aquatic animals in the channels in lower November. Nat. Furutonenuma.

[76] Morino H., Kusano H., Holsinger J.R. (2004). Description and distribution of *Crangonyx floridanus* (Crustacea: Amphipoda: Crangonyctidae) in Japan, an introduced freshwater amphipod from North America. Contrib. Biol. Lab Kyoto Univ..

[77] Cannizzaro A.G., Balding D., Lazo-Wasem E.A., Sawicki T.R. (2019). Morphological and molecular analyses reveal a new species of stygobitic amphipod in the genus *Crangonyx* (Crustacea: Crangonyctidae) from Jackson County, Florida, with a redescription of *Crangonyx floridanus* and notes on its taxonomy and biogeography. J. Nat. Hist..

[78] Cannizzaro A.G., Sawicki T.R., Schaefer J., Castrillo D., Maxwell E.C., Burch E., Culver D.C., Niemiller M.L. (2026). Two new species of *Stygobromus* Cope, 1872 (Amphipoda, Crangonyctidae) amphipods from hypotelminorheic seepage springs in the Washington, DC, USA area. Subterr. Biol..

[79] Andújar C., Arribas P., Yu D.W., Vogler A.P., Emerson B.C. (2018). Why the COI barcode should be the community DNA metabarcode for the metazoa. Mol. Ecol..

[80] Bensasson D., Zhang D.X., Hartl D.L., Hewitt G.M. (2001). Mitochondrial pseudogenes: Evolution’s misplaced witnesses. Trends Ecol. Evol..

[81] Cannizzaro A.G., Lange C.J., Berg D.J. (2023). A new species of stygobitic *Hyalella* Smith, 1874 (Amphipoda: Hyalellidae) from Ash Meadows National Wildlife Refuge, Nevada, USA, with discussion of the unique presence of the species in the Nearctic groundwater fauna. J. Crustac. Biol..

[82] Cannizzaro A.G., Lange C.J., Berg D.J. (2025). Gateways to the underworld: Molecular phylogenetic analyses identify patterns of groundwater fauna and two new species of hypogean *Hyalella* Smith, 1874 (Amphipoda, Hyalellidae) from the northern Mojave Desert. Subterr. Biol..

[83] Libertini A., Rampin M. (2009). A molecular cytogenetic study on some Icelandic amphipods (Crustacea) by fluorescence in situ hybridization (FISH). Open Zool. J..

[84] Beermann J., Westbury M.V., Hofreiter M., Hilgers L., Deister F., Neumann H., Raupach M.J. (2018). Cryptic species in a well-known habitat: Applying taxonomics to the amphipod genus Epimeria (Crustacea, Peracarida). Sci. Rep..

[85] Porter T.M., Hajibabaei M. (2018). Over 2.5 million COI sequences in GenBank and growing. PLoS ONE.

