# Supplementary material for: Assessing the Small Nuclear Ribosomal Subunit (18S rRNA) as a Potential Barcoding Gene for Amphipoda (Malacostraca: Peracarida) Along with the Design of a Unique Primer Set to Target the Amphipod 18S rRNA Gene
Source: Biology (Basel). 2026 Oct 2;15(19):1752. doi: 10.3390/biology15191752 (PMC13664141; doi:10.3390/biology15191752)

Table S1. Comparison of the 18S locus across multiple species to determine the degree of ambiguities (i.e., heterozygosity) within individual sequences and the degree of intraspecific variability. Sequences highlighted bold were produced as part of this study.

Family	Species	Specimen ID	GenBank accession #	# of base pairs	Heterozygosity	Intraspecific variability (#bp)
Crangonyctidae	*Crangonyx apalachee*	TRS 160.1	OM845753	2019	0	0
		TRS 160.2	OM845754	2023	0	0
		TRS 160.4	OM845756	1989	0	0
		TRS 160.8	OM845757	2025	0	0
		TRS 160.9	OM845758	2019	0	0
		TRS 160.13	OM845759	2022	0	0
		TRS 160.14	OM845760	1976	0	0
	Crangonyx stinei	TRS 255.1	OQ703715	1592	0	0
		TRS 255.7	OQ703719	1352	0	0
		TRS 255.9	OQ703721	2108	0	0
	Crangonyx parhobbsi	TRS 91.1	**QB063752**	2107	0	0
		TRS 91.2	**QB063751**	1441	0	0
		TRS 91.4	MT470695	1350	0	0
	Crangonyx grandimanus	TRS 101.1	PQ522380	2095	0	0
		TRS 108.1	MF579390	1628	0	0
		TRS 111.1	**QB063749**	2220	0	0
	Crangonyx manubrium	TRS 30.1	PQ522353	1474	0	0
		TRS 54.1	MH992722	2166	0	0
		TRS 85.6	**QB063748**	1476	0	0
	Crangonyx ephemerus	TRS 191.5	MN460203	2196	0	0
		TRS 191.6	MN460204	1662	0	0
		TRS 191.8	MN460206	1633	0	0
	Crangonyx pseudoephemerus	TRS 191. 3	MN460195	1586	0	0
		TRS 191.10	MN460196	1596	0	1
		TRS 191.11	**QB063756**	1640	0	0
	Stygobromus floridanus	TRS 90.1	MK778478	2162	0	0

		TRS 90.2	MK778477	2158	0	0
		TRS 90.3	MK778474	2157	0	0
	Stygobromus doughertyensis	TRS 207.1	MK778482	1407	0	0
Gammaridae	*Gammarus* nr. *daiberi*	TRS 370.1	PX695420	1206	0	0
		TRS 370.2	PX695421	1496	0	0
		TRS 370.4	PX695422	1592	0	0
		TRS 370.5	PX695423	1499	0	0
	Gammarus cf. *daiberi*	TRS 254.1	PP377630	2010	0	0
		TRS 254.2	PX695414	2028	0	0
		TRS 254.3	PX695415	2182	0	0
		TRS 254.5	PX695410	2021	0	0
		TRS 293.8	PX695416	1897	0	0
		TRS 293.9	PX695417	1906	0	0
	Gammarus mucronatus	TRS 242.1	PX695424	1994	0	0
		TRS 297.1	**QB063744**	2147	0	0
		TRS 297.2	PX695425	2149	0	0
		TRS 297.3	**QB063746**	1491	0	0
		TRS 297.4	**QB063745**	2140	0	0
	Gammarus tigrinus	TRS 350.4	PP377631	1526	0	0
		TRS 350.5	PP377632	1885	0	0
		TRS 250.2	PP377628	1983	0	0
		TRS 250.3	PP377629	2129	0	0
	Gammarus minus	TRS 341.1	PZ274479	1411	0	0
		TRS 341.2	PZ274480	1995	0	0
		TRS 348.3	PZ274482	2110	0	0
		TRS 348.4	PZ274483	2126	0	0
		TRS 367.1	PZ274484	2001	0	0
		TRS 414.1	**QB063753**	1897	0	0
		TRS 414.2	**QB063754**	1894	0	0
Hyalellidae	*Hyalella dentata*	AGC 519.3	PX093674	1470	0	1
		AGC 1188.1	PX093675	1410	0	0

	Hyalella wakulla	TRS 268.1	**QB063760**	2035	0	0
Corophiidae	*Apocorphium lacustre*	TRS 212.2	**QB063755**	1912	0	0
Ampithoidae	*Cymadusa compta*	TRS 245.1	**QB063750**	2010	0	0
Artesiidae	*Artesia subterranea*	AGC 1359.1	**QB063743**	2071	0	0
Hadziidae	*Paraholsingerius smaragdinus*	AGC 888.1	**QB063741**	2002	0	0
Talitridae	*Mexorchestia carpenteri*	AGC 2.1	**QB063739**	2079	0	0
Brevitalitridae	*Talitroides alluaudi*	AGC 14.1	**QB063740**	2113	0	0
Seborgiidae	*Seborgia relicta*	AGC 1331.1	**QB063742**	2099	0	0

Figure S1. Example alignment of the 18S AROH based on selected members of the *Crangonyx floridanus* species complex.

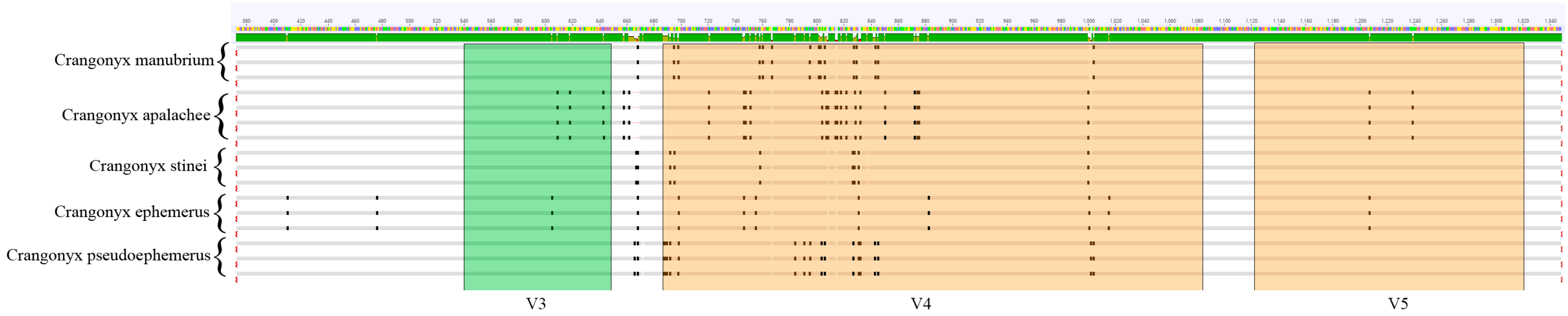

Supplement: Supplementary file 1 [file biology-15-01752-s001.zip › biology-4559396-supplementary.pdf]
